# Stability versus Neuronal Specialization for STDP: Long-Tail Weight Distributions Solve the Dilemma

**DOI:** 10.1371/journal.pone.0025339

**Published:** 2011-10-07

**Authors:** Matthieu Gilson, Tomoki Fukai

**Affiliations:** 1 Lab for Neural Circuit Theory, Riken Brain Science Institute, Saitama, Japan; 2 Core Research for Evolutional Science and Technology, Japan Science and Technology Agency, Tokyo, Japan; 3 The Brain and Neural Systems Team, RIKEN Computational Science Research Program, Saitama, Japan; Neuroscience Campus Amsterdam- VU University, The Netherlands

## Abstract

Spike-timing-dependent plasticity (STDP) modifies the weight (or strength) of synaptic connections between neurons and is considered to be crucial for generating network structure. It has been observed in physiology that, in addition to spike timing, the weight update also depends on the current value of the weight. The functional implications of this feature are still largely unclear. Additive STDP gives rise to strong competition among synapses, but due to the absence of weight dependence, it requires hard boundaries to secure the stability of weight dynamics. Multiplicative STDP with linear weight dependence for depression ensures stability, but it lacks sufficiently strong competition required to obtain a clear synaptic specialization. A solution to this stability-versus-function dilemma can be found with an intermediate parametrization between additive and multiplicative STDP. Here we propose a novel solution to the dilemma, named log-STDP, whose key feature is a sublinear weight dependence for depression. Due to its specific weight dependence, this new model can produce significantly broad weight distributions with no hard upper bound, similar to those recently observed in experiments. Log-STDP induces graded competition between synapses, such that synapses receiving stronger input correlations are pushed further in the tail of (very) large weights. Strong weights are functionally important to enhance the neuronal response to synchronous spike volleys. Depending on the input configuration, multiple groups of correlated synaptic inputs exhibit either winner-share-all or winner-take-all behavior. When the configuration of input correlations changes, individual synapses quickly and robustly readapt to represent the new configuration. We also demonstrate the advantages of log-STDP for generating a stable structure of strong weights in a recurrently connected network. These properties of log-STDP are compared with those of previous models. Through long-tail weight distributions, log-STDP achieves both stable dynamics for and robust competition of synapses, which are crucial for spike-based information processing.

## Introduction

Modifications of the strength (or weight) of synaptic connections between neurons that occur in an activity-dependent manner are hypothesized to play an active role in generating the structure of neuronal networks [1]–[7]. The importance of the relative timing between pre- and postsynaptic spikes for the weight modification, known as spike-timing-dependent plasticity (STDP), has been demonstrated in many brain areas and across many species [8]–[10]. Many models have been proposed to investigate the functional implications of STDP; see [11] for a review. Owing to its time scale, STDP can capture fine temporal correlations between incoming spike trains to select some synaptic input pathways [1], [12]–[16] However, which features of STDP are both biologically realistic and functionally appropriate remains unclear.

In this paper, we propose a novel STDP rule, termed log-STDP, that can produce long-tail distributions of synaptic strengths similar to those reported in recent experiments. Pyramidal cells in the rat visual cortex exhibit lognormal-like distributions for the amplitudes of excitatory postsynaptic potentials (EPSPs) [17]. Electrophysiological measurements in the barrel cortex of mice also revealed rare large-amplitude responses in addition to more frequent medium- and small-amplitude responses [18]. In addition to their long-tail character, the observed distributions also exhibit a couple of outliers many times (e.g., 20) stronger than the mean. Similar long-tail distributions have also been observed by two-photon imaging of dendritic spines in the hippocampal CA1 of young rats [19], where the spine size may be positively correlated with the strength of synapse [20]. These findings led us to investigate the conditions under which STDP can generate such long-tail weight distributions in an activity-dependent manner. While a learning rule leading to lognormal weight distributions was formulated in terms of firing rates [21], spike-based mechanisms have not been examined theoretically. A recent numerical study [22] made use of spread weight distributions obtained using STDP, but did not investigate the underlying dynamics. Here we focus on the conditions allowing STDP to produce long-tail weight distributions.

Moreover, we study the functional implications of log-STDP in terms of synaptic specialization. We focus on how STDP can achieve both a stable weight distribution and effective selection of synaptic input pathways, which we refer to as the stability-versus-function “dilemma”. Additive STDP (add-STDP) can rapidly and efficiently select synaptic pathways by splitting synaptic weights into a bimodal distribution of weak and strong synapses [1], [14], [23]. However, the stability of the weight distribution requires hard bounds due to the resulting unstable weight dynamics. Moreover, even for uncorrelated inputs, add-STDP can split a unimodal weight distribution, in a way that does not meaningfully represent the input statistics. In contrast, weight-dependent update rules can generate stable unimodal distributions [24]–[26]. Weight dependence is supported by experimental observations [27], which have been used to fit the multiplicative STDP (mlt-STDP) proposed by van Rossum et al. [24]. On the down side, weight dependence weakens the competition among synapses and may lead to only weakly skewed weight distributions. Narrow unimodal weight distributions are functionally less interesting than either bimodal or spread distributions with significant positive skewness [22]. Gütig et al. showed that an intermediate parametrization between add-STDP and the multiplicative STDP of Rubin et al. [25] provides a solution to the dilemma [15]; we will refer to their “non-linear temporally asymmetric” model as nlta-STDP. However, their model relies on a “soft” upper bound for synaptic weights and thus is not naturally reconcilable with long-tail weight distributions. We will examine the advantages of log-STDP for 1) representing the statistical properties of input spike trains (i.e., spike-time correlations) [15], [28]–[30] and 2) the reorganization of existing circuitry to adapt to a new input configuration [2], [31]. In doing so, we will compare log-STDP with the “extreme” cases of add-STDP and mlt-STDP, as well as nlta-STDP.

## Results

**Figure 1:**
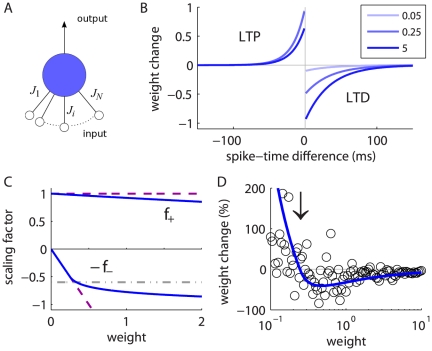
Single neuron equipped with STDP-plastic synapses. **A:** Single neuron excited by 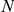 input spike trains. The synaptic strength of synapse 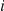 is denoted by 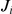. **B:** Potentiation (LTP) and depression (LTD) curves 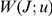 with 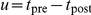 in (4). Darker curves indicate stronger values for the weight 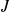: 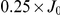 (light blue), 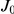 (medium blue), and 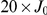 (dark blue) in (6). In the top left quadrant for LTP, the two curves in lighter blue are superimposed, since potentiation is quasi-constant for small weights. **C:** Functions 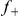 for LTP and 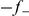 for LTD in log-STDP (blue solid curve) in (6) with 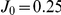, 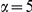 and 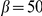; mlt-STDP similar to van Rossum et al.'s model [24] (pink dashed line); and add-STDP similar to Song et al.'s model [1] (gray dashed-dotted curve for depression and pink dashed curve for potentiation). **D:** Weight change (in percent of the original weight) resulting from 20 successive modifications induced by log-STDP with random pairing of pre- and postsynaptic spikes (within the range 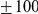 ms). In qualitative agreement with experimental measurements [19], smaller weights experience large fluctuations whereas larger weights exhibit less variability. The mean expected modification (blue solid curve) and 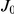 is indicated by the vertical arrow.

We first explain how we derived the novel model of log-STDP. Then, we study the synaptic dynamics for a single neuron whose plastic synapses are stimulated by an arbitrary number of input spike trains, as illustrated in Fig. 1A. Finally, we examine how the results for a single neuron extend to the case of a recurrent network.

### Toy plasticity model producing lognormal weight distribution

**Figure 2:**
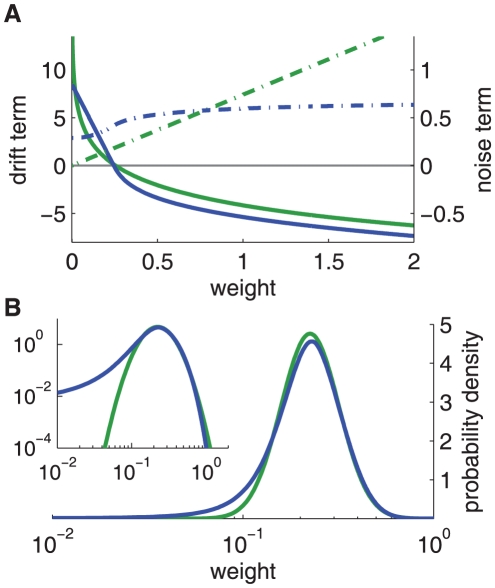
Comparison between the toy model and our new model of log-STDP. **A:** Plot of the functions 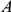 (solid curves, left y-axis) and 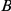 (dashed-dotted curves, right y-axis) that describe the first and second moments of the weight dynamics, cf. (1). Comparison between log-STDP (blue curves) with 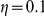, 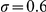, 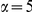, 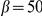, 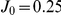, 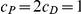, 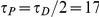 ms in (3), (4) and (6); and the toy model (green curves) with 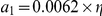, 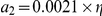, 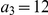, and 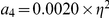 in (22). **B:** Solutions of the Fokker-Planck equation for the curves plotted in A. The x-axis has a log scale. Left inset: log scales for both axes.

Following previous studies [24], [29], [32], we use the Fokker-Planck formalism to study the probability density 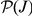 of a population of weights 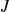 that are modified by many plasticity updates. Denoting by 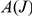 and 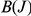 the first and second stochastic moments of the weight updates (or drift and diffusion terms, resp.), the stationary solution of the Fokker-Planck equation is the following distribution:
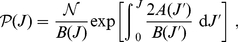
(1)where 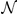 is a normalization factor. We observe that there exists a family of functions 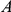 and 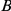 for which the expression in (1) is exactly a lognormal distribution, namely
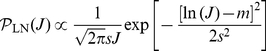
(2)with parameters 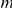 and 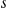, the latter being related to the spread of the distribution. Typical examples for 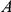 and 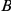 are represented in green in Fig. 2A (solid and dashed curves, resp.). The key features here are the decreasing log-like saturating profile for 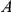 which crosses the x-axis, and the linearly increasing function for 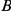. Note that these conditions need only be satisfied around the crossing value to obtain a close-to-lognormal distribution. Details can be found in Methods with explicit expressions for 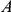 and 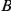 in (22). However, we cannot regard this fictive plasticity model, hereafter referred to as ‘toy model’, as biologically realistic. A first reason is that the mean weight update in the case of uncorrelated inputs is 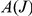, which diverges as the weight 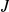 approaches 0. Another reason is that an STDP rule cannot be explicitly derived from this model. For STDP, 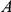 and 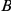 cannot be freely chosen, but are tied to each other. Nevertheless, from this toy model we design a biologically realistic STDP rule that is also inspired by the experimentally-inspired mlt-STDP proposed by van Rossum et al. [24].

### STDP model capable of generating long-tail weight distributions

Here we present the mathematical description of ‘log-STDP’. In this phenomenological model, the change in the synaptic weight induced by pre- and postsynaptic spikes at respective times 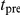 and 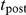 is given by
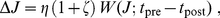
(3)where the learning rate 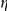 determines the speed of learning. The Gaussian white noise 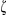 describes the variability observed in physiology; it has zero mean and variance 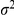. Here, we treat the case where all spike pairs contribute to STDP. Depending on the relative timing of the spike pair 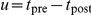, the learning window 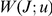 represented in Fig. 1B leads to potentiation (LTP) or depression (LTD), respectively:
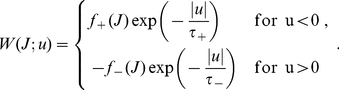
(4)


The shape of the weight distribution produced by STDP can be adjusted via the scaling functions 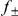 in (4) that determine the weight dependence. These functions are involved in the drift term 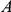 and noise term 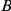 that determine the synaptic dynamics and particularly the stationary weight distribution in (1). For a general model of STDP described by (3) and (4) 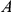 and 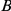 are given by:
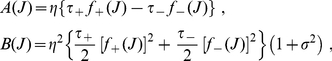
(5)where 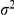 is the variance of the white noise 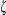. The derivation of (5) neglects input-output correlations. This is a good approximation when a neuron is stimulated by many uncorrelated inputs. In this case, the neuron model does not play a significant role in the synaptic dynamics. Details can be found in Methods (‘STDP dynamics for uncorrelated inputs’). Here the idea is to obtain similar dynamics for the toy model and the STDP rule, such that the latter produces lognormal-like weight distributions. To do so, we match the functions 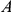 (solid curves) and 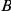 (dashed curves) for our novel model (blue) and the toy model (green) represented in Fig. 2A. In particular, we focus on the profile of 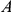 around its crossing point with the x-axis to infer the shapes of the LTP and LTD curves. From (5), 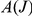 relates to the difference 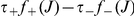. To obtain the log-like profile of 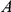 in the toy model, several possibilities can be imagined. An option is increasing LTP and linear LTD, somewhat similar to the ‘power-law’ STDP model proposed by Morrison et al. [26]. However, we will focus on the “converse” solution with almost constant LTP and sublinear LTD. This leads to the following expressions that are represented in Fig. 1C:
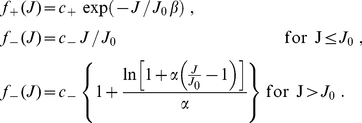
(6)LTD discriminates between the ranges of small weights (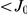) and large weights (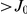). The weight dependence for LTD in log-STDP is similar to mlt-STDP [24] for 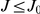, i.e., it increases linearly with 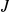. However, the LTD curve 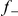 becomes sublinear for 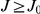, and 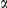 determines the degree of the log-like saturation. This choice is motivated by examining the sole effect of changing LTD for “large” weights compared with the classic model of mlt-STDP. In practice, we choose the function 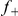 for LTP to be roughly constant around 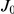, such that the exponential decay controlled by 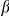 only shows for, say, 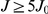. Note that, in the range 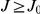, log-STDP coincides with mlt-STDP when 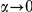 and 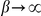; and it tends toward add-STDP when 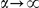 and 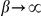.

### Noise scheme

Before studying the dynamics induced by log-STDP, we discuss the role of noise in our model in the light of previous models. Our model involves two sources of noise in the STDP dynamics, via the white noise 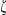 (with variance 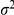) and the learning rate 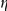 in (3). The learning speed resizes the weight updates, which matters when input spike trains are random to a large degree. As can be seen in (5), the order of magnitude between 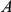 and 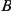 crucially depends on 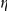 [29]. Because 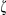 modulates the term involving 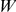 in (3), its effect depends on 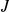 via the scaling functions 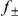. For log-STDP with quasi-constant LTP and sublinear LTD, the noise experienced by a strong weight is weaker *in proportion* as compared to a weaker weight; see Fig. 1D. In this sense, log-STDP qualitatively resembles the model of activity-dependent plasticity used by Yasumatsu et al. [19] to explain the observed fluctuations of spine volumes. In contrast, the original model proposed by van Rossum et al. [24] involves a STDP noise that linearly increases with the weight 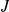 for both LTP and LTD, namely 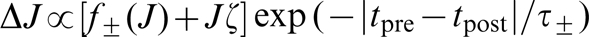. Further details are discussed in Methods (‘Baseline parameters for log-STDP’).

Compared to the study by van Rossum et al. [24], we use a relatively fast learning rate 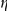 and a weaker value for 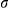 in our version of mlt-STDP (and log-STDP, etc.). The original model of van Rossum et al. assumes that the variability observed in the weight updates [27] originates from STDP only. There, the intrinsic variability of single synapses and measurement noise are neglected. This means that STDP updates may not be as noisy as proposed by van Rossum et al. This motivates the use of a smaller value for 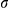 here. Note that, interestingly, plasticity-independent variability has been recently reported to be proportionally larger for weak than strong synapses [18]. This goes in the same line as more stability for strong weights in our model, via the dependence of 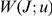 on 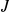.

A last point concerns spike-pair restrictions: all pairs of pre- and postsynaptic spikes contribute to STDP in the present study, which implies more updates and thus more noise in the synaptic dynamics. Consequently, even though individual updates in our version of mlt-STP are less noisy than in the original model of van Rossum et al. [24], the global noise experienced by the synaptic weights is comparable in both models during the ongoing spiking activity and leads to spread distributions.

### Predicting the stable weight distribution

Our theoretical framework allows us to evaluate the weight distribution produced by an arbitrary weight-dependent STDP model, by combining (1) and (5). In this section, we focus on the case of uncorrelated input spike trains, for which (5) is valid. However, the theoretical prediction may not be reliable when the synaptic dynamics does not have a stable fixed point. For example, add-STDP requires taking into account the effect of input-output spike-time correlations to obtain a bimodal distribution of [24], [32]. Such theoretical refinements will be discussed later. In this study, 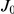 is chosen such that LTP and LTD in log-STDP (roughly) balance each other for uncorrelated inputs, namely 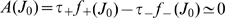. It corresponds to the intersection of the drift (solid curve) and the x-axis in Fig. 2A. Therefore, 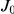 will also be referred to as the ‘fixed point’ of the dynamics in the following. In the absence of noise and for slow learning, the weights cluster around the fixed point 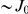, when it is stable (negative slope for 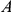). Otherwise, the weight distribution spreads around the fixed point. The noise term 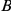 (dashed curves in Fig. 2A) can be somewhat interpreted graphically from the LTP and LTD curves, 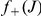 and 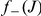 in Fig. 1C. When they are farther apart, the resulting noise is stronger. In log-STDP, because depression increases sublinearly (blue solid curve for 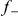 in Fig. 1C), noise in log-STDP is weaker than that for mlt-STDP for which depression increases linearly (pink dashed curve for 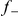). Figure S1 provides a qualitative comparison of the relationship between the 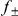 curves (column A) and the drift and noise terms (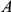 and 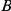 in column B) for different STDP models, as well as the resulting weight distributions (column C).

**Figure 3:**
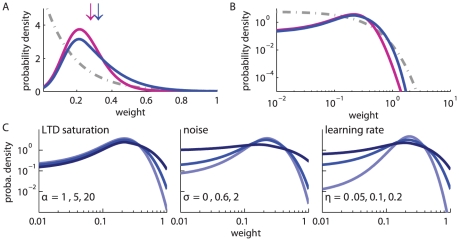
Theoretical predictions of weight distribution shaped by STDP. **A:** Resulting weight distribution for log-STDP (blue solid curve) with the saturation for LTD corresponding to 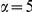 in (6); mlt-STDP inspired by the model of van Rossum et al. [24] (pink solid curve) in (27); and add-STDP [1], [14] (gray dashed-dotted curve) in (26). Log-STDP and mlt-STDP are parameterized to obtain roughly the same equilibrium value for the mean weight (arrows); without noise and very slow learning, the resulting narrow distribution would be centered around the fixed point 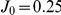. The curves are evaluated using (1) and (5) with the same learning rate 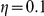 and noise level corresponding to 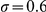 in (3). **B:** Similar to A with log-scaled axes. **C:** Effect of the parameters in log-STDP. Comparison between the predicted weight distributions with the baseline parameters 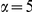, 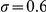 and 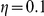 in (6) (medium blue curve in B) and two variants with the parameter change indicated in each plot (darker curves correspond to larger values).

As a first control, we verify that the stationary distributions in Fig. 2B are similar for the toy model and log-STDP, even though we only roughly match 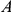 and 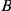 in Fig. 2A. The tail of strong weights vanishes slightly faster for log-STDP than for the toy model (see inset with a log-log plot) because of the weaker noise for large weights, cf. the dashed curves in Fig. 2A. The comparison with mlt-STDP (pink solid curve) in Fig. 3A shows the influence of sublinear LTD. The weight distribution is more skewed and the tail of large weights extends further for log-STDP (blue solid curve); see also Fig. 3B with log-scaled axes. Even though the difference between log-STDP and mlt-STDP may not look dramatic in Fig. 3A and B, we will show later that the underlying dynamics are clearly different, especially in the case of correlated inputs. The weight distribution for add-STDP (gray dashed-dotted curve) is spread because our choice of parameters leads to strong noise in the synaptic dynamics (especially the fast learning rate 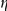). Note that, in contrast to Fig. 3B, STDP can also lead to a bimodal distribution clustered at each bound or even a unimodal distribution located at the upper bound, e.g., for weaker LTD than used here. Then, the value of the upper bound on the weights may critically affect the resulting distribution.

The toy model is sufficiently simple to obtain an analytical expression for the spread of the resulting distribution, see (24) in Methods. Because of the proximity between the dynamics induced by the toy model and log-STDP, we can predict the effect of the parameters in log-STDP on the stationary weight distribution. These trends are illustrated in Fig. 3C (log-log plots), which compares the weight distributions for the baseline parameters (medium blue curve; same as Fig. 3B) and two variants for a given parameter, a smaller value and a larger value (lighter and darker blue curves, resp.). For larger 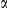, LTD has a more pronounced saturating log-like profile and the tail of strong weights extends further. Both stronger noise with a larger value for 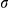 and a faster learning rate 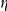 strengthen the shuffling of the weights, which results in more widely spread distributions.

### Continuous shuffling of synaptic weights

**Figure 4:**
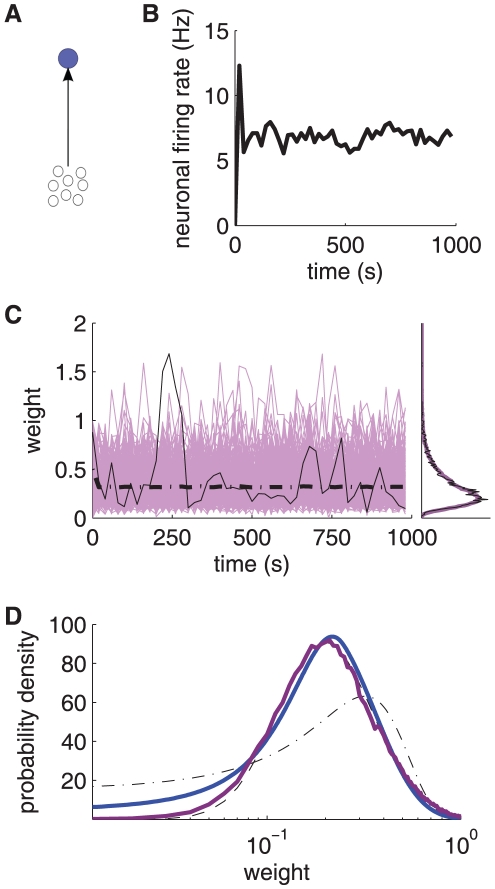
Strong shuffling of individual weights within the stable distribution. **A:** Schematic diagram of the neuron (top blue filled circle) stimulated by a pool of 3000 uncorrelated inputs (bottom open circles). **B:** Evolution of the neuronal output firing rate. **C:** Evolution of synaptic weights in the case of uncorrelated inputs. The purple traces represent a portion of the input weights recorded every 20 s, the black thin trace corresponds to an individual weight, and the black thick dashed-dotted trace indicates the mean weight over the 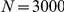 inputs. Right inset: The mean weight histogram averaged over the learning epoch is plotted in purple and the spot histogram at time 1000 s is represented by the black thin line. **D:** Comparison between the simulated weight distribution (purple thick curve; it corresponds to the purple curve in C) and the analytical prediction (blue thick curve; same as Fig. 3) with a log scale for the weights (x-axis). The black thin curves represent a Gaussian fit (dashed-dotted) and a lognormal fit (dashed) of the simulated weight distribution, obtained using linear regression. The same baseline parameters as in Fig. 3 have been used here.

Rapid adaptation to the external world is enhanced when weights experience a certain degree of noise. With log-STDP, synapses are shuffled because of the plasticity-intrinsic noise 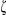 and random input spikes in a highly dynamical manner, even after the synaptic population reaches the equilibrium state. To show this, we conduct numerical simulations of an integrate-and-fire neuron (parameters are given in Methods) with 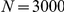 synapses, each receiving uncorrelated (Poisson) spike trains with input firing rate 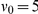 Hz (Fig. 4A). The output neuronal firing rate, hereafter denoted by 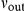, stabilizes between 6 and 8 Hz (Fig. 4B). The evolution of synaptic weights is displayed in Fig. 4C, which shows that individual synaptic weights are constantly shuffled by STDP (cf. black thin trace) within the stable weight distribution (right inset). The simulated mean weight (black thick dashed-dotted trace) stabilizes around 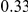, which is actually larger than the fixed point 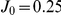: this mainly follows because of the lower bound enforced on the weight at 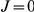, which prevents the weights from spreading downward. (The solution of the Fokker-Planck equation takes this into account via the boundary condition at zero.) In Fig. 4D, the resulting weight distribution (purple curve) is satisfactorily predicted by expression in (1) (blue curve), except for small weights. The latter discrepancy arises from the finite size of the weight updates. Two fits using linear regression on the simulated weights (black thin curves) confirm that their distribution is closer to lognormal (dashed curve) than Gaussian (dashed-dotted curve). Figure S2 provides comparisons between the simulated and predicted distributions when varying the parameters 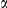 and 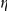. Those simulation results agree with the predictions in Fig. 3C.

### Representation of input spike-time correlations in the weight structure

**Figure 5:**
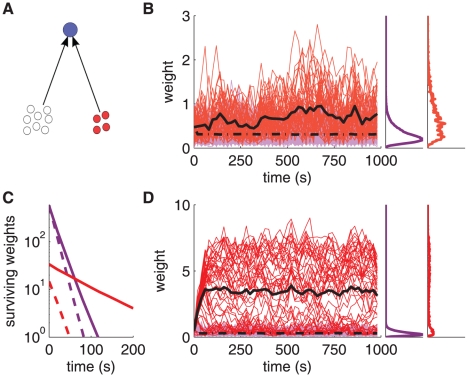
Input spike-time correlations lead to robust weight specialization. **A:** Schematic diagram of the neuron (top blue filled circle) stimulated by a pool of 2950 uncorrelated inputs (bottom open circles) and a pool of 50 correlated inputs (bottom red filled circles). **B:** Evolution of the synaptic weights for the configuration in A with weak correlation (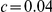). The correlated group is favored (red traces, mean in black thick solid line), synonymous with a greater chance of appear in the tail of the distribution compared to uncorrelated inputs (purple traces, mean in black thick dashed-dotted line). Right insets: normalized time-averaged histograms. **C:** Survival time of the weights from uncorrelated (purple curves) and correlated (red curves) inputs in the top 20% of the distribution. Comparison between log-STDP (solid curves) and mlt-STDP (dashed curves). The y-axis indicates the number of synapses present in the top 20% at each counting round (performed every 20 s) from 100 s until the time on the x-axis. The data are averages over 20 trials. **D:** Similar to B with stronger correlation (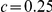). The weights from correlated inputs are pushed out of the main body of the distribution and saturate to a much larger value than the mean (black thick dashed-dotted line), roughly 30 times larger here.

The temporal “antisymmetry” (i.e., LTP versus LTD) of the learning window has been shown to favor correlated inputs, therefore generating weight specialization [1], [14], [15]. In order to examine how an input correlation structure is encoded in the weight structure by STDP, we consider the configuration in Fig. 5A that involves a small group of correlated inputs (bottom red circles) among many other uncorrelated inputs (bottom open circles). The correlated group consists of 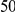 input spike trains that have instantaneous pairwise spike-time correlations with strength 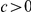. The mean firing rate is the same for uncorrelated and correlated inputs, namely 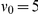 Hz. Details about the input generation can be found in Methods (‘Generating correlated spike trains’). Only a few tens of inputs take part in the volleys of correlated spikes, which are embedded in the synaptic bombardment of the total 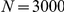 inputs. In comparison, in the absence of any other stimulation, the coincident spiking of more than 500 inputs is necessary to trigger an output spike. In this sense, we consider “weak” spike-time correlations in a physiologically plausible range.

When the inputs are only weakly correlated (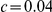, meaning that 20% of the spikes are involved in synchronous events for each input), the weight distribution remains unimodal, as illustrated in Fig. 5B. Nevertheless, weights from correlated inputs are found more often in the tail of the distribution (red traces). In Fig. 5C, the weights from correlated inputs (red solid curve) survive for a longer time in the top 20% of the distribution compared to uncorrelated inputs (purple solid curve). The mean dwell time for both groups of inputs is given in Table 1. (Note that the “survival” here does not consider the history of the weights between the checks that are performed every 20 s. Nevertheless, this describes well the comparative trends in the persistence of strong weights for the different STDP models.) Weights from uncorrelated inputs are subject to shuffling only, whereas weights from correlated inputs also experience (weak) potentiation. Although the inputs remain correlated, the temporary weight structure is not robustly sustained and is erased due to the STDP noisy dynamics.

**Table 1:** Mean dwell time of the input weights in the top 20% of the distribution.

	uncorrelated inputs	correlated inputs
log-STDP	9.0 s	78.4 s
mlt-STDP	5.2 s	11.6 s

The dwell times correspond to the simulation for a single neuron and weak input correlation in Fig. 5A–C.

In contrast, stronger input correlations (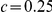, meaning that 50% of the input spikes correspond to synchronous events) can potentiate the corresponding weights to a value many times larger than the mean. In Fig. 5D, the mean weight for the 50 correlated inputs is 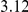 (with the strongest weights up to 10), as compared to 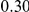 for the 2950 uncorrelated inputs. Here the drift clearly overpowers the noise to extract those weights from the main body of the distribution. Strongly potentiated weights are inhomogeneous and experience relative stability despite the noise (see the black trace of an individual weight). This occurs even for identical synaptic delays, meaning that the weight potentiation is not all-or-nothing, but rather gradual.

When synaptic inputs involve multiple correlated groups, log-STDP can sort the corresponding mean weights in increasing order of their correlation strengths; see Fig. S3 for an illustrative example. Both the slowly increasing LTD and decaying LTP contribute to this effect. The trends shown here are in agreement with previous results using the almost-additive version of nlta-STDP and the Poisson neuron model [30], which examined in depth the potentiation for several input pools with distinct correlation levels and different degrees of weight dependence. Note that nlta-STDP incorporated single-spike plasticity contributions in order to sort the mean weights of the input groups depending on their correlation strengths between the lower and upper weight bounds in that previous study. Here, however, log-STDP may produce a multimodal weight distribution, but the global mean of the distribution is kept small (around 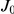). Therefore, the weights from strongly correlated inputs are pushed to the tail of strong synapses while the majority of weights remains in the main body of weak synapses. The emerging distribution may thus be highly skewed.

### Sensitivity to input correlations

**Figure 6:**
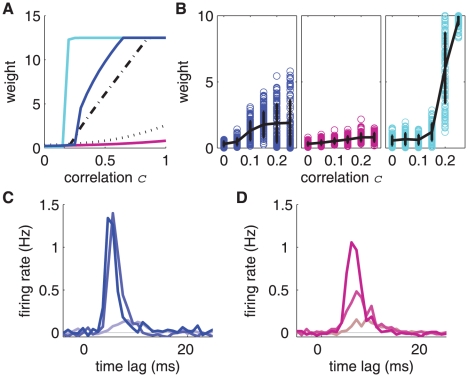
Sensitivity to input correlations. **A:** Theoretical equilibrium weights plotted as a function of the input correlation. Comparison of log-STDP (blue), mlt-STDP (pink), nlta-STDP (cyan), Hennequin et al.'s model [22] (dashed-dotted black) and ‘power-law- STDP of Morrison et al. [26] (dotted black). The parameters for the Poisson neuron and the last two models are the same as Fig. S1. The curves are estimated using the zeros of (20), which is based on the Poisson neuron and also neglects the effect of noise. **B:** Simulated potentiated weights as a function of the input correlation for the same configuration as in Fig. 5B and D. Comparison of log-STDP (blue), mlt-STDP (pink) and nlta-STDP (cyan). The respective mean weights and standard deviations are represented by the black curves and error bars. The results are taken from 10 simulations. **C & D:** Post-stimulus time histograms of the output neuron after training (averaged over 100 s), where the stimuli are the input correlated events. Comparison between **C** log-STDP (blue) and **D** mlt-STDP (pink) for 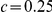, 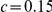 and 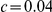 (from darker to lighter color, resp.). For log-STDP, the neuronal response is reliably and precisely triggered by correlated events (more than 80% occurrence) for 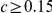, whereas mlt-STDP yields 70% for 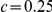 and only 50% for 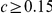.

Now we examine in more detail how log-STDP is sensitive to input correlations. For any STDP model, potentiated weights imply stronger input-output correlations and, in turn, larger LTP induced by STDP. This self-reinforcing potentiation mechanism may be blocked when the weight dependence is “too” strong, though. Because of its sublinear profile for LTD and the resulting spread weight distribution, log-STDP exhibits an enhanced potentiation capability compared to mlt-STDP. Using the Poisson neuron model, we can evaluate how the equilibrium mean weight for the correlated inputs depends upon the input correlation 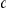. This provides a qualitative prediction for the behavior of integrate-and-fire neuron, for which a full calculation is out of the scope of this paper. Figure 6A illustrates the predicted effect of input correlations for several STDP models; see (21) in Methods for details on the calculations. Log-STDP (blue curve) exhibits a rather steep curve for the fixed point, indicating graded but strong potentiation when input correlations increase. For comparison, we examine the model recently proposed by Hennequin et al. [22], which has a roughly piecewise profile for LTD with a slower increase for 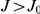 than 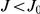 (the details are provided in Supporting Information). Because of this change in curvature, this model behaves similarly to log-STDP (black dashed-dotted curve). The nlta-STDP model proposed by Gütig et al. [15] is also sensitive to input correlations. In the parameter range where nlta-STDP can induce strong potentiation (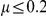 in (28) in Methods), the equilibrium weight always exhibits a sharp step from the lower to the upper bound (cyan curve). Outside this parameter range, nlta-STDP resembles mlt-STDP, meaning weak competition. In other words, potentiation for nlta-STDP is rather all-or-nothing. In contrast to these three models, mlt-STDP (pink curve) and power-law STDP proposed by Morrison et al. [26] (black dotted curve) appear far less sensitive to input correlations. LTD in both models increases linearly with the weight, which strongly counterbalances LTP. The weak potentiation of correlated inputs by mlt-STDP explains the only minor increase of stability for the tail of the distribution in Fig. 5C (thick dashed curves) and Table 1. The weight distributions corresponding to the five STDP models are illustrated in Fig. S1 (column C). Although the predictions in Fig. 6A do not include noise, simulations in Fig. 6B for log-STDP (blue), mlt-STDP (pink) and nlta-STDP (cyan) agree with the trends. Namely, log-STDP exhibits a gradual potentiation of correlated inputs, which is intermediate between the weak increase for mlt-STDP and the all-or-nothing behavior for nlta-STDP. The number of correlated inputs also plays a role here: a larger correlated group induces stronger potentiation (as indicated by (18) in Methods), as does stronger correlation.

The presence of strong weights also affects the neuronal output firing rate. The simulation for log-STDP in Fig. 5D corresponds to 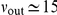 Hz (black solid line in Fig. S3C). In comparison, the baseline firing rate for uncorrelated inputs stabilizes around 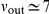 Hz in Fig. 4B. The larger total incoming weight in Fig. 5D alone does not explain the gap in the firing rate. Rather, this significant increase arises because input correlated events cause the neuronal output to effectively fire. This is confirmed by the post-stimulus time histogram of the output neuron in Fig. 6C, where correlated events are taken as the reference stimulus. The stronger input correlations are (indicated by darker color), the stronger some weights are potentiated and the more reliable the drive of the output firing by each correlated event is. For 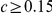, the neuronal response is locked to each input correlated event with log-STDP. In Fig. 6D, mlt-STDP (darker to lighter pink) leads to a weaker and later-in-time histogram, especially for 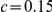 (medium pink). The corresponding neuronal firing rate is then 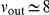 Hz, almost unchanged compared to about 7 Hz for uncorrelated inputs. These results clarify that the neuronal response is robustly and precisely driven in a broader range of input correlations for log-STDP than for mlt-STDP. Note that the good overall reliability of the neuronal response even when weights are weakly potentiated (especially for mlt-STDP) is partly related to the integrate-and-fire neuron model. The difference between log-STDP and mlt-STDP is much clearer when using a Poisson neuron as shown in Fig. S5, for which the output firing probability linearly increases with the synaptic weights.

**Figure 7:**
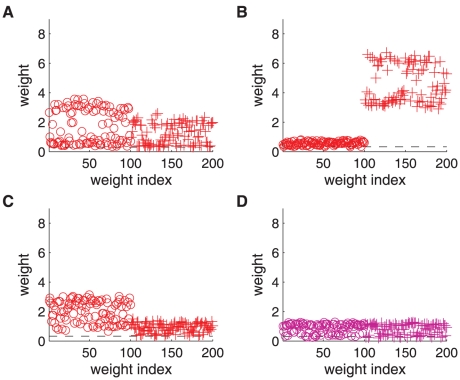
Competition between two identical strongly correlated input pools. The configuration is similar to Fig. 5 with two groups of 100 correlated inputs each with the same strength 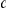, in addition to 2800 uncorrelated inputs. **A:** Winner-share-all for 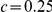 and log-STDP with 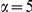. **B:** Winner-take-all for 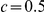 and log-STDP with 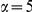. **C:** Winner-take-all for 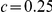 and log-STDP with 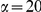. **D:** Winner-share-all for 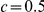 and mlt-STDP. The circles and pluses indicate the weight strengths averaged values over 300 s of simulation (after the initial development of the structure) for the two correlated groups, respectively. The dashed line indicates the fixed point 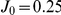 of the weight dynamics.

Now we show how the sensitivity to input correlations for log-STDP and mlt-STDP (Figs. 6C and D) affects the resulting synaptic competition. When two identical correlated groups (with no correlation between each other) excite a neuron, a desirable outcome is the specialization to only one of those while discarding the other. This is important to select functional pathways in a consistent manner, without “mixing” spiking information. Add-STDP and nlta-STDP can perform such a ‘symmetry breaking’, whereas mlt-STDP cannot do so [2], [15]. Because of its sensitivity to input spike-time correlations shown in Fig. 6C, we expect log-STDP to be capable of symmetry breaking, at least when input correlations are sufficiently strong. For the baseline parameters (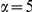) and strong correlations (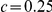), the first correlated group slightly dominates (circles), but does not completely repress the other group (pluses) in Fig. 7A. However, with very strong correlations (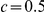) in Fig. 7B, the second group clearly takes over the driving of the neuronal firing, and the red group is at the level of uncorrelated inputs (black dashed line). With still 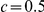, but tuning LTD closer to mlt-STDP with 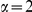, we obtain a similar situation to that in Fig. 7A, with no clear winner (not shown). In such winner-share-all cases, either group may slightly and temporarily dominate the other group during the simulation (and roles may swap over time), but both groups coexist in the tail of strong weights. In contrast, winner-take-all can be obtained for 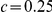 as in Fig. 7A when using a more pronounced saturating LTD (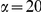), as illustrated in Fig. 7C. Altogether, stronger saturation for LTD and, to a lesser extent, stronger potentiation (i.e., higher values for 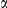 and 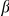 in our model, resp.) favor a winner-take-all behavior. In contrast, the same simulation as Fig. 7B with mlt-STDP not only shows weakly potentiated weights, but the two input groups cannot be separated by the learning dynamics; only a winner-share-all behavior occurs (Fig. 7D).

### Remodeling of synaptic pathways

**Figure 8:**
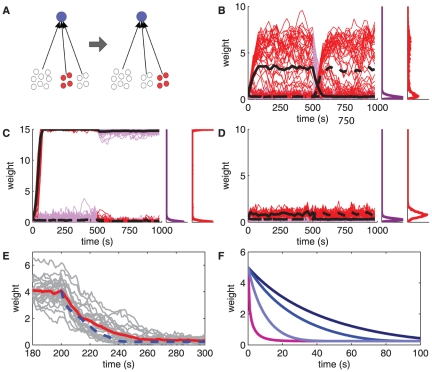
Remodeling of synaptic weights when the input configuration changes. **A:** Schematic representation similar to Fig. 5A for two groups of 50 inputs each, which exhibit strong spike-time correlations (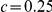, represented by the bottom red filled circles) only between 0 and 500 s for the first group and between 500 and 1000 s for the second group. **B,C,D:** Comparison of the evolution of the synaptic weights for **B** log-STDP (baseline parameters); **C** add-STDP with a ratio between depression and potentiation equal to 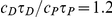 and an upper bound set to 15; and **D** mlt-STDP where depression is linearly increasing with the current value of the weight strength (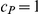 and 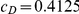). These three plots are similar to Fig. 5B, except that red traces indicate weights coming from correlated inputs only when correlation is turned on (purple otherwise). The black thick solid and dashed curves represent the respective mean weights for the first and second correlated groups, respectively. **E** Decay of potentiated weights back to the baseline equilibrium value (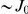) after input correlation is switched off. Simulated weights are represented by gray traces and their mean by the red thick curve. The theoretical prediction in (7) is plotted in dashed-dotted blue. **F** Comparison of the predicted decay of potentiated weights for mlt-STDP (pink) and log-STDP for 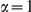, 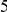 and 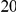 (light to dark blue, resp.). The curves correspond to (7), where 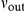 is calculated using the Poisson neuron model.

The external world to which the brain has to adapt keeps changing over time. When the input configuration changes significantly, a desirable behavior for a neuron with plastic synapses consists in forgetting the previously learned weight structure to readapt. To compare the performance of the different STDP models, we consider a neuron receiving inputs from a large uncorrelated pool and two small pools (either uncorrelated or correlated) of 50 inputs. As illustrated in Fig. 8A, the two pools switch their correlation strengths at 500 s: before 500 s the first (second) group is strongly correlated (uncorrelated), while after 500 s the second (first) group is strongly correlated (uncorrelated, resp.). The restructuring process goes quite efficiently with mlt-STDP (Fig. 8D), but not with add-STDP (Fig. 8C). Because of unstable weight dynamics, add-STDP may fail to forget the previously learned structure [31]. The strong weights clustered at the upper bound then drive the neuronal output (even without input correlations), which prevents the second correlated group to be learned. The stronger the upper bound, the more difficult it is for the neuron to readapt. In contrast, even though mlt-STDP manages to readapt, the weight specialization remains weak, as explained in the previous section. Because of its well-balanced dynamics, log-STDP successfully combines the strong points of add-STDP and mlt-STDP. As shown in Fig. 8B, log-STDP rapidly selects the input pathway from the second group when it starts to show strong correlations, while rapidly weakening the pathway from the first group. Note that similar results can be obtained with nlta-STDP.

After the correlation switch at 500 s, the potentiated weights from the first correlated group return to their baseline equilibrium value, close to the fixed point 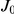. In a similar simulation to that in Fig. 8B, the weights stronger than 1 at 500 s are represented by the gray traces in Fig. 8E. Their decay is driven by the drift 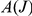, which is affected by the weight dependence [31]. Neglecting noise, we can use the expression in (5) to approximate the trajectory of the mean weight (black curve)
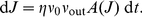
(7)By integrating this formula and using the simulated firing rate for 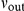, we obtain the blue dashed-dotted curve, which satisfactorily predicts the decaying mean weight. From (7), it is clear that a weaker drift 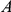 leads to a longer decay time. In Fig. 8F, a more pronounced saturating LTD (i.e., larger values for 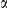) increases the decay time, up to several tens of seconds. In comparison, mlt-STDP (pink curve) forgets the learned structure after a much shorter period. (The trajectory for mlt-STDP is exponential [31], but a simple analytical result cannot be derived for log-STDP. The Poisson neuron model was used to evaluate 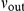.)

### Emergence and persistence of a weight structure in a recurrently connected network

In order to assess whether the interesting dynamics produced by log-STDP for a single neuron also holds in the case of a recurrent network, we first reproduce a previous result of network self-organization [33]. The goal for STDP is to split of the initially homogeneous distribution for both input and recurrent weights. As shown in Fig. 7, such a symmetry breaking requires strong competition. As illustrated in Fig. S6, log-STDP produces a clear weight structure that represents the input correlation configuration, even though the potentiation is weaker than in Fig. 5D. Here log-STDP performs as well as an almost-additive version of nlta-STDP model in terms of competition.

**Figure 9:**
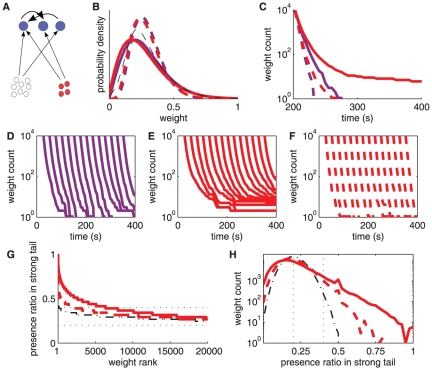
Stability of the emerging structure of strong weights in a recurrently connected network. **A:** Schematic representation of the network with plastic recurrent connections (thick arrows) and fixed input connections (thin arrows). The network neurons (top blue filled circles) are excited by one pool of 2900 uncorrelated inputs (bottom open circles), and one pool of 100 inputs (bottom red filled circles) whose spike trains may be correlated. **B:** Time-averaged distributions of the recurrent weights over the learning epoch. Comparison of log-STDP (solid curves) and mlt-STDP (dashed curves) when the small group (red filled circles in A) is uncorrelated (purple) and correlated (red). **C:** Survival of strong synapses (top 20%) of the distribution over time. The color coding is similar to B. As in Fig. 5C, checks are performed every 5 s and the y-axis indicates the number of surviving synapses from 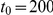 s until the time on the x-axis, cf. (8). **D,E,F:** Similar curves to B with different starting times 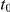. Comparison of **D** log-STDP with no correlation; **E** log-STDP with correlations; and **F** mlt-STDP with correlations. **G:** Ratio of presence in the top 20% at each check (every 5 s between 200 and 395 s) for the initially strongest at 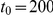 s, cf. (9). Comparison between log-STDP (solid curve) and mlt-STDP (dashed curve) for correlated inputs. The weight indices (x-axis) are sorted. The two horizontal dotted lines indicate 20% and 40%, respectively. **H:** Distributions of the presence ratio corresponding to G, with a log-scaled y-axis.

Following the results in Fig. 5C, we evaluate now whether log-STDP favors the stability of strong weights in a network. As illustrated in Fig. 9A, the network neurons have plastic recurrent connections (thick arrows) and fixed input connections (thin arrows) from two pools of inputs, here 2900 with no correlation (open circles) and 100 with correlations (red filled circles). To compensate the partial connectivity (10% for all connections), all inputs have a higher firing rate equal to 10 Hz and the input weights have been scaled up (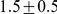) in order to obtain neuronal firing rates in the same range as in the case of a single neuron (Fig. S7). Even without input correlation, recurrent excitatory connections induce (positive) spike-time correlations. The cross-correlograms between neurons are symmetric [33], which results in both LTP and LTD. Due to a net LTD effect, the weight distribution in Fig. 9B is slightly shifted toward smaller values (purple thick solid curve), compared to the case of feed-forward connections (black thin dashed curve). Here input correlations have a small effect on the weight distribution, as indicated by the red solid curve in Fig. 9B to be compared with the purple solid curve. The resulting interneuronal correlations are weak and comparable to the situation in Fig. 5B.

However, these input correlations do affect the fine structure of recurrent connections for log-STDP. To show this, we firstly examine the “survival” of the potentiated synapses in the top of the distribution, as in Fig. 5C. Figure 9C represents the survival of the strongest synapses from time 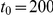 s onwards, checks being performed every 5 s. The curves correspond to the number of weights that are present in the top 20% of the distribution at each check from 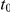 to 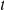, in a similar fashion to Fig. 5C. Formally, we denote by 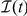 the set of weight indices in the top 20% of the whole population at time 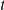 (roughly 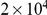 among the total 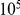 synapses). The curves in Fig. 9C correspond to the number of weights in
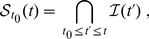
(8)where 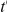 is a multiple of 5 s. When the small pool of 100 inputs has no correlation, the number of surviving synapses in 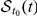 decreases to zero (purple solid curve). In contrast, correlated inputs allow strong synapses to survive for a longer time (red solid curve) and a few even persist until the end of the simulation. Figure 9D and E show similar curves for different starting times 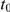. For uncorrelated inputs, the surviving time is comparable for all 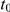 and no structure emerges. However, input correlations build up a structure (Fig. 9E), which grows larger as time goes.

Compared to log-STDP, the weights are shuffled more quickly with mlt-STDP and no structure develops. This is illustrated in Fig. 9C by the thick dashed curves, to be compared with the thick solid curves. The survival time of strong weights for correlated inputs with mlt-STDP (red dashed curve) is even shorter than that for uncorrelated inputs with log-STDP (purple solid curve). The mean dwell time for the 6000 weights that last the longest in the top 20% is given in Table 2. Note that only a few recurrent weights persist in the tail for a long time compared to the input weights of a single neuron (leading to smaller values compared to Table 1), because the correlations between network neurons are quite weak here.

**Table 2:** Mean dwell time for the 6000 recurrent weights that last the longest in the top 20% of the distribution.

	without input correlation	with input correlation
log-STDP	7.2 s	9.0 s
mlt-STDP	4.5 s	5.0 s

The dwell times correspond to the simulations of the recurrent network in Fig. 9C. Because roughly two thirds of the 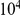 initial weights in the tail (top 20%) at 200 s disappear at the following counting round, only the 6000 weights with longest dwell times are taken into account here.

Finally, we assess the persistence of weights in the strong tail in another manner. Because input correlations are sustained here, it makes sense to check how many times each weight appears in the strong tail. The repeated presence of weights in the tail implies some consistency for an emerged weight structure, even though some weights get repressed and pushed out at some times. We thus calculate for each weight 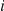 the ratio of presence in the strong tail between 200 and 395 s (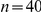 checks), namely
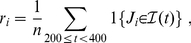
(9)where 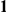 is the characteristic function, valued 1 when its argument is true. The 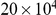 highest ratios 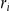 are plotted in Fig. 9G in a rank order for log-STDP (red solid curve) and mlt-STDP (red dashed curve) when inputs have correlations. The (smoothed) histograms of 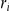 for the whole population is represented in Fig. 9H. As expected, we find more weights with a higher ratio 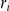 for log-STDP than mlt-STDP, meaning that the tail of strong weight is more stable over time. In the extreme case where the synaptic dynamics is very noisy, the weights in the strong tail are like chosen by a random draw of 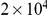 weights among the total 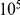. Here it corresponds to the average presence ratio 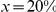 (lower horizontal dotted line in Fig. 9G) and the standard deviation 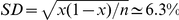, as a random draw of a portion 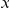 of elements within the whole pool 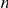 checks. We set a significance threshold for the ratios 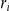 at three times the standard deviation above the mean (the upper horizontal dotted line in Fig. 9G indicates 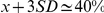). For a random draw every 5 s (thin dashed-dotted curve), only 130 weights among the total 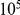 have a ratio 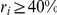. With mlt-STDP, 2142 weights satisfy 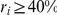, but only 46 weights 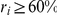. This is much lower than the figures for log-STDP, for which about a third of the tail, namely 6351, have 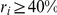 and 1075 weights 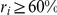. The same calculations with the 10% strongest weights for the tail instead of the 20% give similar results.

## Discussion

**Figure 10:**
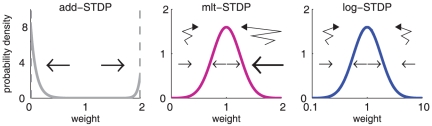
Schematic illustration of stability and neuronal specialization. Comparison of additive STDP; multiplicative STDP; and our new model of “lognormal” STDP (*log scale* for the weights on the x-axis). The horizontal arrows represent the direction of the weight drift resulting from STDP for different values of the weight; a thicker arrow indicates a stronger drift. For mlt-STDP and log-STDP, the zig-zag arrows represent the noise, whose amplitude is indicated by the horizontal scaling.

The present paper proposes a novel STDP model called log-STDP that combines a number of interesting properties. Log-STDP inherently produces long-tail (e.g., lognormal-like) distributions of synaptic strengths that agree with physiological observations [17], [18]. From a functional point of view, log-STDP combines the strong points of add-STDP and mlt-STDP: robust specialization and flexibility, respectively. A schematic comparison of their synaptic dynamics is given in Fig. 10. Two main ingredients underly the desirable properties of log-STDP: 1) a sublinear weight dependence for LTD and 2) noise in the STDP update that spreads the weight distribution, but does not shuffle strong weights too strongly compared to weak weights.

### Weight dependence and noise scheme

A first important feature of log-STDP is its log-like saturating LTD, an intermediate variation between constant and linear functions. The scaling functions in (6) have been designed to coincide with mlt-STDP model in the range of “small” weights (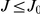). This choice was motivated by studying the effect of the change from linear to sublinear LTD for 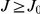. One could argue that extremely strong synapses are less likely to be observed in physiology (even though easy to detect). Consequently, saturation of LTD for strong weights may not appear clearly in available data, such as those [27] used to fit van Rossum et al.'s original model [24]. (Here we have chosen 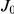 to be both the point where the curvature for LTD changes and the fixed point of the learning dynamics. If the range where LTD is linear extends beyond the fixed point, the main body of the weight distribution and dynamics will resemble those for mlt-STDP, while the properties of log-STDP would only be observed if some weights can become larger than 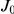.) Although we have formulated a direct relationship between the weight and LTD here, recent experiments in hippocampal microcircuits have shown that LTD (and LTP) for excitatory synapses can be regulated by GABAergic signals in a way that depends on the excitatory weight [34]. Such functional network effects appear compatible with our model of saturating LTD (personal communication).

In addition, LTP decays slowly for large weights in our model. Such a decrease for LTP can be related to a limitation of resources at the synaptic site, such that the weight does not grow indefinitely. For very strongly correlated inputs, this is important in order to prevent a runaway behavior of the weights (results not shown). Similar to mlt-STDP and in contrast to add-STDP and nlta-STDP, log-STDP requires neither “hard” or “soft” upper bound on the weights to secure the stability of their distribution.

Another property of log-STDP that supports its functional capabilities is the noise in the STDP update. Due to the sublinear LTD (and quasi-constant LTP), 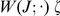 grows more slowly than 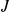 in magnitude. It follows that large weights experience less variability in proportion to their current value than small weights (Fig. 1D). Here we have considered noise in the weight update only; a further step consists in incorporating activity-independent noise in the synaptic strengths. For example, recorded EPSPs exhibit a large variability [18] or, on a slower time scale, spine volumes fluctuate even when NMDA receptors are blocked [19]. Interestingly, such fluctuations were found to be smaller proportionally to their mean for larger synapses. This means less relative variability for strong synapses, in line with our model.

The present analysis only considers all-to-all spike contributions to STDP. For low (input and output) firing rates, as was used here, typical interspike intervals are larger than the temporal range of STDP. This means that the synaptic dynamics for models with restricted interactions, where not all pairs of spikes contribute to STDP [35], [36], is practically the same as in our (unrestricted) case. For high firing rates, such restrictions imply fewer updates and thus less noise in the weight dynamics. Nevertheless, the Fokker-Planck calculations adapted to spike-pair restriction lead to similar expressions to (5); see Supporting Information for the example of input-restricted STDP. We thus expect our results to qualitatively hold in general (e.g., influence of saturating LTD). Similar results were obtained using the alternative parametrization for sublinear LTD in (25) in Methods, and with the Poisson neuron model (although this requires stronger input correlations, see Supporting Information for details). This suggests that our conclusions mainly arise from the qualitative properties of log-STDP, but do not heavily rely on fine tuning or a specific neuron model.

### Shaping the weight distribution

Because of its sublinearly increasing LTD, log-STDP alone produces a long-tail weight distribution, even for uncorrelated inputs. The change of curvature around the fixed point of the dynamics (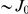 in our model) is a key factor to spread the tail of strong weights (Fig. 3C). Intrinsic noise in the STDP updates and fast learning also contribute to spread the weight distribution. Weights from correlated inputs are pushed toward the tail of weight distribution. Saturating LTD and decreasing LTP lead to graded equilibrium values for weights in terms of the corresponding correlation strengths (Figs. 7A, B and S3B). Without being so dramatic a case compared to binary synapses [37], log-STDP can produce a clear structure where some weights (Fig. 5D) or all weights (Fig. 7) from correlated groups are separated from the main body of the distribution. A more elaborate input structure with inhomogeneous correlation levels is expected to modify the tail of strong weights. For example, graded input correlations lead to graded potentiation that further populates the tail of the distribution (Fig. S3). A recent study [22] has used gradually correlated inputs (repeating spike pattern) in order to obtain a long-tail distribution without noise in the STDP update. This was made possible using a change of curvature for LTD (quasi piecewise-linear curve) in the triplet STDP model [38] around the fixed point for the weight dynamics. In any case, we stress that log-STDP produces a long-tail distribution for a broad range of input configurations. The resulting distribution is compatible with the data obtained by Song et al. [17] and Lefort et al. [18]. For example, when sampling a “small” number (say, a few hundreds) of weights from those in Fig. 5D or Fig. S3, the resulting distribution has a lognormal-like main body together with a few very strong outliers.

### Functional implications

Activity-dependent plasticity in general and STDP in particular aims to represent the statistical properties of the input spike trains in the weight structure. Here we have focused on the case where spike-time correlations dominate the synaptic dynamics. For correlated inputs, log-STDP performs a selection of input pathways close to the performance of add-STDP [1], [14]. As an example that requires strong competition, Fig. S6 shows symmetry breaking in a recurrently connected network for both afferent and recurrent connections [33]. Depending upon the input configuration and log-STDP parameters, both winner-take-all and winner-share-all behaviors may occur (Figs. 7 and S3). This is important in the context of spike-based independent component analysis (symmetry breaking in Fig. 7B being the simplest example), for which winner-take-all is necessary [2], [7], [39]. Log-STDP exhibits strong competition for large values for the parameter 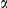, as nlta-STDP does for small values of the power factor 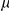. The competition appears more gradual with log-STDP, though (Figs. 6A and B). Specifically about nlta-STDP, beyond the biological relevance of the soft upper bound, an issue concerns whether the bound takes similar values or differs across synapses. Various bounds can lead to a spread tail in the weight distribution, but would imply “unfair” competition between synapses (i.e., some would be easier to potentiate). With log-STDP, all synapses experience the same dynamics and their potentiation level thus reflects the input correlations, leaving aside the noise. On the other hand, log-STDP with small values for 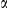 resembles mlt-STDP, which appears clearly inferior in terms of synaptic competition. Note that the stronger STDP noise in the original model of van Rossum et al. [24] further impairs the neuronal specialization, especially for weak spike-time correlations. Although we have constrained our study to the case of pools of coincidentally firing inputs, these conclusions are expected to hold for any inputs with correlations in the temporal range of STDP, such as spike patterns [23]. Additional mechanisms such as synaptic scaling may be used, for example, to constrain the neuronal firing rate in a homeostatic fashion. In our model, adjusting the fixed point 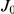 (e.g., decrease when the output firing rate is “too” high) would guarantee that the flexibility and robustness of our results are preserved. Our results were obtained using axonal delays; the effect of synaptic delays on the topology and persistence of weight structure is left to subsequent study.

When the input configuration changes, synaptic weights trained by log-STDP rapidly reorganize to adapt to the new configuration pattern (Fig. 8B). This rapid rewiring is also favored by the continuous shuffling exhibited by the individual synapses receiving uncorrelated inputs (Fig. 4B). Note that the newly learned inputs are very strongly potentiated, as if learned from scratch. In other words, the previously learned structure is completely forgotten (after 50 s in Fig. 8B). This arises from the intermediate parametrization between add-STDP and mlt-STDP, in a similar manner to nlta-STDP [15].

A last point concerns the stability of the emerged weight structure. Sufficiently strong input correlations is necessary to overcome the relatively strong noise used here. The presence of strong weights has been shown to be useful for pattern activity [4], firing avalanches [40], and spike-based information transmission [22]. In such cases, the stability of the tail of strong weights is crucial for sustaining the spiking activity in a consistent fashion over time. During the stimulus presentation, so long as the drift of the weights dominates the synaptic dynamics, the stability of the learned structure is ensured (Figs. 5D and 8B). In contrast, for weak correlations, noise may be comparable to the drift in Figs. 5B and 9. This implies a competition between shuffling and sustained potentiation of the weights. Then, our model of noise in log-STDP turns out to be crucial to favor the stabilization of a weight structure. Even the weak spike-time correlations that arise within a recurrent network stimulated by a rather small number of correlated inputs can be picked up by log-STDP to build up among plastic recurrent weights a structure that can persist over a significant period (hundreds of seconds in Fig. 9). In contrast, mlt-STDP induces too strong a shuffling, which prevents such a structure to emerge and stabilize. After the end of the stimulus presentation, the persistence of potentiated weights determines the memory depth of the learning system. After ceasing the stimulus presentation, the decay time back to the baseline level is longer for more pronounced LTD saturation in log-STDP (larger value for 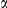 in Fig. 8F), generalizing previous results for add-STDP and mlt-STDP [31]. Altogether, weaker LTD and noise for large weights improve their stability.

### Conclusion

Our results show that weight dependence and noise in the weight update are crucial features to obtain a realistic and functionally efficient STDP model. To our knowledge, this has not been explicitly studied in biophysical models of STDP [7], [41], [42]. In complement to previous studies on weight-dependent STDP [15], [24], [25], [29], we have focused on the advantages for STDP to generate long-tail distributions that involve weights many times stronger than their mean. In our model, the extent to which weights are potentiated is determined by the interplay between the STDP properties (LTD profile) and input correlations (group size and correlation strength). The tail of strong weights encodes the “meaningful” component of input statistics and gives rise to function (e.g., temporal correlation transmission). In this way, log-STDP overcomes the limitations of mlt-STDP when synapses have (roughly) linear responses. Our results open a promising way to investigate persistent synaptic structures and efficient spiking information processing in neuronal networks.

## Methods

Using a mathematical model of STDP, we examine the relationship between the weight dependence and the resulting learning dynamics. First, we present a framework to study the synaptic dynamics based on the Fokker-Plank formalism. This allows us to study the stationary weight distribution for various STDP models. Then, we study particular solutions of the Fokker-Planck equation that are exactly lognormal distributions. This family of solutions is referred to as ‘toy model’, from which log-STDP is derived. Finally, we provide details on the parameters used in the present study.

### Fokker-Plank formalism

We constrain the theoretical analysis to the case of a single neuron excited by an arbitrary number 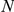 of synapses, cf. in Fig. 1A. Following previous studies [24], [32], [36], we adapt the framework to the model of STDP defined by (3) and (4), for which all pairs of pre- and postsynaptic spikes contribute. The Fokker-Planck equation determines the evolution over time of the probability density 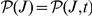 of the synaptic weights. When the weights are modified by many STDP updates, they can be assimilated to transitions in the state space 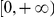. Denoting by 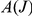 and 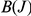 the first and second stochastic moments of the weight updates, respectively (or drift and diffusion terms), the general formulation is given by
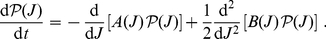
(10)Equating the lhs of (10) to zero leads to the unique normalized solution in (1), which is the stationary distribution. To study (1), it is necessary to evaluate the functions 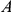 and 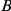. As their names imply, 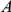 describes the mean effect (first stochastic moment) and 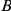 the variability (second moment) of the weight update 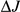 in (3):
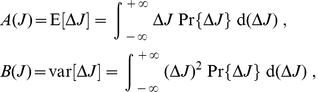
(11)where 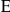 and 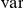 denotes the expectation value and the variance, respectively. Following (3) in the main text, the probability 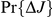 of a given value for the weight update 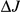 depends on the probability of two independent factors: the respective timing of the pre- and postsynaptic spike trains for each synapse (denoted by 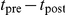), and the Gaussian white noise 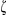. This leads to
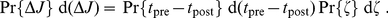
(12)Equation (12) means that the integration with respect to 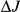 in (11) can be performed by integrating with respect to the two independent variables 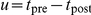 and 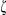 over the real line (for each of them). In our model, the probability density 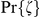 is a Gaussian function with zero mean and variance 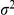. Then, the probability 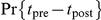 is the key quantity to calculate the drift term 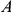 and noise term 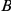.

### STDP dynamics for uncorrelated inputs

In this section, we focus on a simple solution for (12), assuming that the following conditions are satisfied:

- The pre- and postsynaptic spike trains are (quasi) probabilistically independent for all pairs input/output; this is a good approximation in the case of many uncorrelated Poisson-generated inputs.
- The neuronal output firing rate is not too high (e.g., 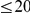 Hz) such that, for each input, an output spike does not effectively interact with too many incoming spikes.

The first point (i) leads to approximated expressions that do not take the neuron model into account, but describe satisfactorily the asymptotic weight distribution when the learning dynamics has a stable fixed point [36]. This means that 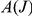 in (5) satisfies 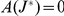 and 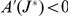 for a given 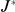. In other words, weight dependence scheme with stronger LTD and/or weaker LTP for larger weights is sufficient, which is the case for log-STDP, mlt-STDP and nlta-STDP. However, add-STDP is weight independent and thus does not satisfy this; its case will be studied in the next section.

Under assumption (i), the pre- and postsynaptic spike trains behave as two Poisson processes. This means that (12) can be rewritten as
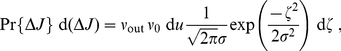
(13)where 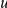 is the spike-time difference, 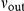 the output neuronal firing rate, and 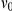 the input firing rate (assumed to be identical for all inputs).

Using (13), the drift 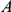 in (11) can be rewritten as:
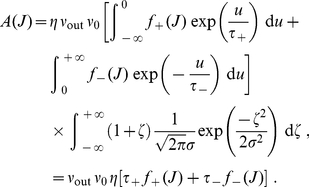
(14)Here we have separated the effect of LTP for 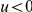 and LTD for 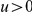, and integrated with respect to the spike-time difference 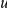. Because the stochastic noise 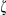 has a zero expectation value, it vanishes in the expression for 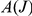.

Likewise, we can evaluate the noise term 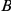 in (11) by replacing the weight update by its square in the integral:
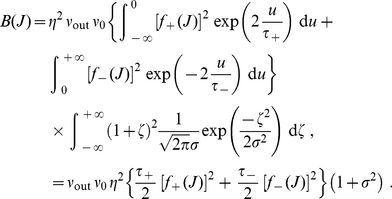
(15)In contrast to the expression for 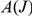, 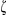 contribute to 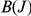 via its variance 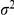. In the previous calculation, it is assumed that the weight changes at each time only concern a single 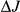 for a single pair of spikes. This is not strictly rigorous: for example, when all pairs of spikes contribute to STDP, a postsynaptic spike may lead to several updates 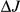 with several input spikes, all contributions being summed together to modify the weight 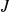. If this does not matter for 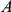, it is problematic for 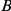 since the square of a sum is not the sum of the squares [36]. Nevertheless we will stick to this approximation assuming relatively low firing rates, in which case not many significant STDP updates occur for each input or output spike.

The results in (14) and (15) are reproduced in (5) in the main text. There, we have dropped the input firing rate 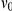 and the output firing rate 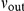, the latter depending on the whole weight distribution. Actually, they do not play any role in the solution in (1) in the case of uncorrelated inputs. Recall that these calculations are valid for any weight dependence 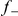 and 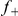, provided the model is formulated using (3) and (4). Although the stability of the stationary solution in (1) is not always granted, this is the case when 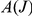 has a stable fixed point for reasonable levels of “noise” 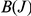 [29].

### Generating correlated spike trains

To obtain a group of spike trains with a given correlation strength 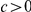, we use a thinning of Poisson processes. More precisely, for each input, the spikes are generated using sampling from two homogeneous Poisson processes [15], [29]. The first process is individual for each correlated input. Its baseline firing rate is set to 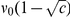. The second ‘reference’ Poisson process is common to all inputs forming a correlated pool and determine correlated events that occur at rate 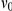. At each correlated event, the concerned inputs increase their instantaneous firing rate such as they take part in the synchronous spike volley with probability 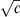. In this way, we obtain a spike train with mean firing rate 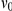 and the desired pairwise correlation strength.

### Taking spike-time correlations into account using the Poisson neuron model

Now we extend the result in (14) and (15) to incorporate input-output correlations. We do not aim to develop the full theory here for integrate-and-fire neuron. Rather, we aim for a simpler result using the Poisson neuron. This provides insight on sensitivity of STDP rules to input spike-time correlations for integrate-and-fire neuron (Fig. 6A). The firing mechanism for the Poisson neuron is governed by a stochastic rate intensity 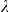, from which spikes are generated as an inhomogeneous Poisson process. Here we consider the simple expression for 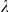, which can be seen as the soma potential and evolves due to the incoming spikes:
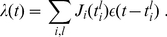
(16)Here the synapse 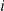 receives inputs at times 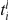 and has weight 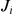. The postsynaptic response kernel 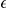 determines the time course of the soma potential for each incoming pulse at synapse 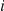; we require 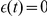 for 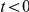, 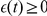 for 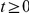 and 
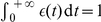
.

When a correlated event occurs, the synchronous incoming spike volley causes the firing probability 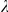 of the neuron to increase on the short time scale of the PSP response. With our model of correlated inputs (see previous section), a given input in a group of size 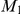 with correlation strength 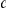 has probability 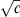 of taking part in any correlated event. Correlated events occur randomly with rate 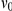. For a given event at time 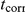, the mean increase of 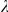 compared to its baseline value 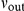 comes from the firing of 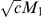 inputs, namely 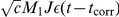. Here we have assumed that the (baseline) expected instantaneous firing rate 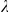 is stationary and can be approximated by 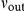, and that homogeneous weights equal to 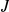 for all inputs from the correlated pool. Outside correlated events, the input spikes come from a spike train with rate 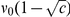 and the probability of spike-time difference is 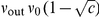 as in (13).

We take input spikes as references now to evaluate 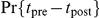. Either an input spike is isolated or it belongs to a correlated event. Summing all contributions, we obtain
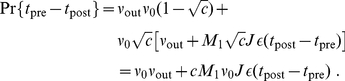
(17)These shortcut calculations are similar to our more general framework that evaluates input-output spike-time covariances [30]. Compared to uncorrelated inputs, the expression in (14) is now augmented by the term involving 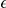 in (17). Because of causality of the neuronal response, the extra term only contribute to LTP. Focusing on the integration over 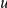 as in (14), this yields
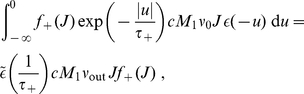
(18)where 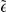 is the Laplace transform of the post-synaptic response kernel 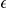; we have used 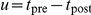. The Laplace transform comes from our use of an decaying exponential function of 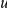 for each side of the STDP learning window 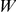 (it would yield a convolution with the corresponding function of 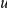 otherwise). Using (16), the mean output firing rate for the Poisson neuron is given by
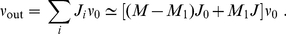
(19)We can thus rewrite the expressions for 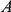 and, likewise, 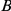:
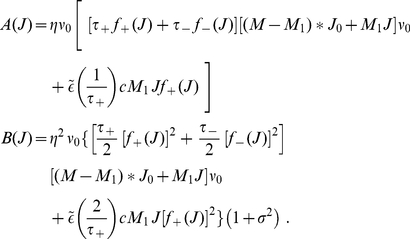
(20)


In particular, the equilibrium weight 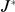 for a single correlated input group of size 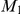 embedded in a total of 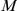 inputs (e.g., Fig. 5D) is given by the zero of 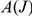, namely
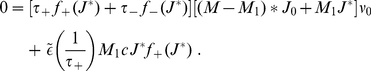
(21)


Using the expressions in (20), Fig. S1 illustrates the effect of input correlations on the weight distribution. This figure gives a qualitative picture of the relationship between the curves of 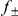 (in A) and the drift and noise terms (in B) on the one hand; and the influence of correlations on the resulting weight distribution on the other hand (red versus gray curves in C). The curves in Fig. 6A represent the fixed point in (21) as a function of the correlation 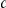 for the different models of STDP. Fig. S4 compares the theoretical prediction in (21) with simulation results using the Poisson neuron model. Last remark, in order to obtain a bimodal distribution for add-STDP, the effect of single spikes on the output firing has to be incorporated. In (20), this amounts to replacing 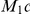 by 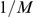.

### ‘Toy plasticity model’ given by lognormal solutions of the Fokker-Planck equation

In order to get analytical insight about a suitable STDP model that generates long-tail distribution of synaptic weights, we consider the following functions:
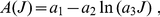
(22)

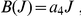
with 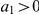, 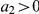, 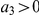 and 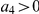. Using these functions as the drift and noise terms in (10), the corresponding solution in (1) becomes
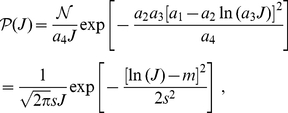
(23)where 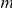 and 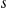 absorb the parameters:
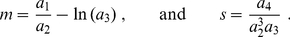
(24)The rhs in (23) is actually the expression for a lognormal distribution with parameters 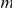 and 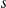, reproduced in (2) in the main text. In particular, the parameter 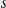, which controls the spread of the distribution similar to the variance for a Gaussian distribution, increases with 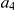 and decreases with 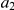 and 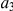.

This toy model of “plasticity” inspired us to formulate the weight dependence for log-STDP in (6). Despite the discrepancies between the functions 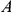 and 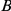 in Fig. 2A, the distributions generated by the toy model and the STDP model are in good agreement, as illustrated in Fig. 2B. Therefore, the effect of the parameters in log-STDP upon the spread of the weight distribution can be inferred from the effects of 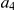, 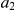 and 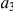 on 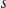 in (24). In function 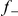, 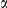 determines the degree of saturation of 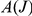 in (5) as 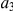 does in (22): larger values imply more pronounced log-like saturation. Likewise, 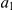 and 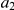 can be related to 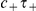 and 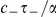, respectively, cf. (5). Altogether, 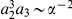, so larger value for 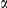 is expected to spread the weight distribution. In (22), 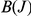 corresponds to a noise whose amplitude is proportional to 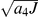. Such a sublinear noise is weaker than the multiplicative noise used by van Rossum et al. [24] and implies smaller variability for larger weights compared to weaker ones. From (3), the noise 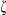 is scaled by amplitude of the noiseless update, which is determined by 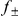. Because of our choice for weight dependence, the resulting noise is weaker than in the toy model. Namely, 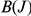 in (5) is 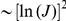 to be compared with (22) for large values of 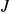; see also Fig. 2A. Both the noise variance 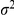 and the learning rate 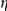 play a similar role to 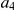: larger values lead to a more spread weight distribution.

### Baseline parameters for log-STDP

The STDP model is detailed in the main text in (3), (4) and (6). The baseline simulation uses 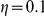; 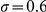; 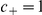; 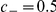; 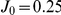; 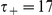 ms; 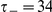 ms; 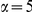 and 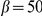. The time constants 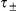 for 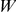 correspond to typical values [27]. For the weight dependence, the scaling functions 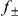 in (6) are chosen such that the equilibrium mean weight is roughly 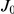 in the absence of noise and for slow learning. To do so, we require the drift 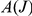 to have a stable fixed point 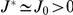, as illustrated in Fig. 1. We thus use parameters such that 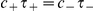, together with 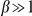.

A previous study [29] has shown that a “fast” learning rate 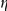 induces noise in the weight dynamics, which can spread the distribution of plastic weights via strong shuffling, compared to “slow” learning. Our choice 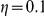 such that a typical weight update is of the order 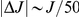 around the equilibrium value for the mean weight. The Gaussian random variable 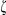 that models the variability of the weight update has zero mean and variance 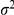. Its standard deviation is chosen 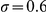, such that the vast majority (95%) of spike pairs corresponding to 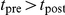 effectively induces depression. This contrasts with the study in van Rossum et al. [24] where 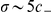, meaning that only around 60% of pairing cases supposedly leading to depression effectively do (i.e., the high level of noise changes the sign of the weight update). In their scheme, however, contributions to STDP were restricted to the nearest presynaptic spike only, which implies fewer updates hence weaker shuffling. In our model, the relatively fast learning rate is also a important source of noise. (As was pointed out to us, our noise scheme cannot be achieved using an implementation of STDP based on cumulative exponential traces [11, Fig 1], for which several weight updates are lumped together; in other words, a noise term cannot be applied to the individual contribution for each pair of spikes in that case.)

### Alternative parametrization for LTD in log-STDP

Similar results were obtained with the following log-like LTD scaling function 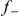 that has a simpler expression:
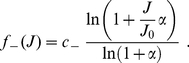
(25)This rule is different from van Rossum et al.'s model [24] for small weights, but it also leads to a fixed point close to 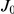, when LTP is roughly constant (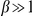).

### Comparison with other models of STDP

In our analysis, we compare log-STDP with other previous reference models, namely add-STDP [1], [14], mlt-STDP [24] and nlta-STDP [15]. This study focuses on the influence of weight dependence on the synaptic dynamics. Therefore, all models follow the equations (3) and (4); they only differ through the scaling functions 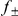. Below, we give the parametrization of 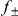 for the other models, to be compared with (6).

Add-STDP [1], [14] is weight independent:
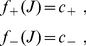
(26)with 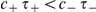 such that LTD overpowers LTP. The drift due to random spiking activity thus causes the weights to be depressed toward zero, which provides some stability for the output firing rate. In numerical simulations, we use 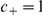 and 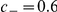, which gives a slightly more unbalanced ratio between LTP and LTD than in Song et al. [1]; this follows because a fast learning rate is used here, synonymous a high level of noise, and more stability thus requires stronger depression.

Mlt-STDP has a linear weight dependence for LTD and constant LTP [24] that was inspired by experimental data [27]:
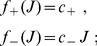
(27)the equilibrium mean weight is then given by 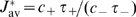. We have 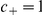 and 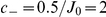 in Fig. 3 such that mlt-STDP and log-STDP coincide for 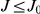. However, simulations in Figs. 5, 8 and 9 were performed using 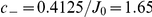, meaning slightly weaker depression than in Fig. 3. This calibration corresponds to a similar neuronal output firing rate to that for log-STDP in the case of uncorrelated inputs.

Nlta-STDP [15] uses a parameter 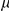 to scale between add-STDP (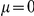) and multiplicative STDP proposed by Rubin et al. [25] (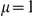):
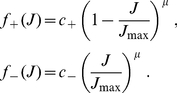
(28)In numerical simulations, the “soft” upper bound is 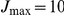, the 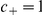 and 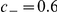. We also set 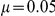 to obtain an almost-additive version of nlta-STDP, such that it leads to strong competition.

### Integrate-and-fire neuron model

The simulation results presented in this paper use the usual leaky integrate-and-fire neuron model with conductance-based synapses. The evolution of the membrane potential 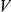 follows the differential equation:
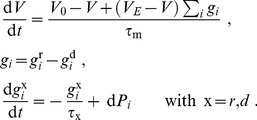
(29)The resetting and resting potential is 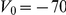 mV, the membrane time constant 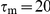 ms, and the reversal potential 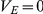 mV. The synaptic influx 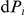 for synapse 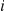 corresponds to a jump (delta function) at each incoming spike after an axonal delay of 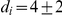 ms; the size of the jump for the conductance strength 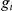 is determined by the synaptic weight 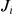 in this paper. The rise and decay time constants for the conductance are 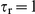 ms and 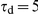 ms. When the threshold 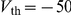 mV is reached, the neuron fires an output spike and 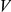 is reset to 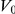 for a refractory period of 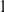 ms, before evolving again due to the presynaptic activity.

## Supporting Information

Figure S1
**Comparison of the weight dependence schemes and resulting weight distributions for different models of STDP.** Each row corresponds to the model whose name is written on the left: log-STDP for our novel model; mlt-STDP [24]; Gütig et al.'s model [15]; Hennequin et al.'s model [22]; and Morrison et al.'s power-law model [26]. **Column A:** 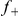 and 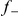 functions that determine the weight dependence (top and bottom, respectively), similar to Fig. 1B. **Column B:** The drift 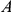 and 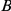 are represented by the solid and dashed curves, respectively (similar to Fig. 2A). The gray curves correspond to the expressions for uncorrelated inputs in Eq 5 in the main text. The red curves represent the drift 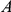 for homogeneously correlated inputs with 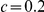 and 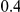 in Eq 20 (from lighter to darker red, respectively); other parameters are 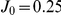; 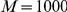; and 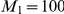. The curves for 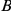 are not shown as they actually are superimposed with the grey dashed curves. **Column C:** Resulting weight distribution (same color coding as B) with linear axes (similar to Fig. 3A). Note that the parameters have not been jointly tuned to obtain, e.g., the same mean weight.(EPS)

Figure S2
**Simulated weight distribution obtained for various choices of parameters for log-STDP.** Similar plots to Fig. 4D in the main text where one of the parameters (indicated above each subplot) differs from those used in the baseline simulation: weaker saturation for LTD with 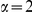; stronger saturation with 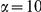; slower learning with 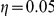; and faster learning with 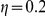. The baseline simulation (thin blue dashed curve) corresponds to Fig. 4D in the main text. The discrepancies between the simulated curve (purple solid line) and the theoretical prediction (black solid line) concerning the range of very small weights relates to the finite size of the weight update. For the range of medium and large weights, on which we focus, the prediction is satisfactory except for the cases of weak saturation (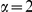) and slow learning (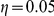). These two simulations actually exhibited a low output firing rate for the neuron, which induced a weak shuffling of the whole distribution of weights; therefore, the solution of the Fokker-Planck equation becomes less accurate.(EPS)

Figure S3
**Simulation with a single neuron similar to** **Fig. 5** **in the main text with two correlated input pools.** **A:** Schematic diagram of the neuron (top blue filled circle) stimulated an uncorrelated pool (bottom open circles) and two correlated pools (bottom red filled circles). Darker gray indicates a stronger correlation strength. **B:** Evolution of the synaptic weights for the configuration in A. The correlation strengths are 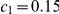 (traces and histogram in lighter red, mean in thick dashed line) and 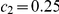 (in darker red, mean in thick solid line), respectively. The plot is similar to Fig. 5B. Our STDP model selects the input pathway with the strongest correlations, but more mildly potentiates the weights coming from that with weaker correlations. **C:** Evolution of the neuronal output firing rate for the configurations: Fig. 5B in dashed-dotted curve; Fig. 5D in dashed curve; and Fig. S2B in solid curve. The emergence of the strong weights results in a significant rise of the neuronal output firing rate, here around 15 Hz to be compared to 5 Hz for uncorrelated inputs in Fig. 4B.(EPS)

Figure S4 **Weigth potentiation and firing rate as a function of the input correlation with the Poisson neuron.** **A:** The single Poisson neuron is stimulated by 1000 inputs of which 100 have correlations (strength on x-axis). The simulated weights from the correlated pool (circles) are taken from 10 simulations of duration 500 s and their mean is indicated by the thick black curve (with error bars for the standard deviation). The predicted equilibrium weights using Eq 21 in the main text is represented by the blue curve. The predictions neglect the noise in the STDP update, as well as the synaptic delays, but it is satisfactory up to correlation strengths equal to 0.6. Discrepancies come from neglecting the noise, which become non-negligible for large weights. **B** Similar to A with the output firing rate.
(EPS)

Figure S5
**Time histogram of the neuronal spiking response to correlated events for a Poisson neuron.** Comparison between log-STDP (red) and mlt-STDP (purple). The parameters given in Sec ‘Parameters used in numerical simulation with the Poisson neuron model’ above. Similar to Fig. 6C and D in the main text, darker colors correspond to stronger input correlations with 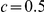, 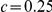 and 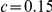. Among the 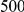 input spike trains, 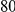 are correlated; all input firing rates are equal to 5 Hz. The difference between log-STDP and mlt-STDP is more pronounced for the Poisson neuron because the output firing probability increases linearly with respect to the input weights. In comparison, the LIF neuron in Fig. 6C and D is in a regime where the sensitivity to correlated inputs is higher; consequently, even the small increase of the weights for mlt-STDP still leads to a significant drive of the neuronal output firing.(EPS)

Figure S6
**Weight emerged structure in a network stimulated by two independent correlated pools.** **A:** Schematic representation of the network before (left) and after (right) the learning epoch. The recurrent network of 500 neurons was stimulated by a pool of 2800 uncorrelated input spike trains (not shown) and two identical correlated pools of 100 spike trains each (bottom red circles), which exhibited delta correlations (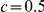). The connectivity probability was 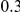 for all input connections and 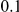 for recurrent connections. All input firing rates are equal to 10 Hz. The equilibrium value 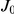 in our STDP model was chosen equal to 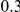 and 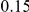 for input and recurrent weights, respectively; the same learning rate was used for both weight sets. Initially, both sets of weights were homogeneous (with 10% randomness). At the end of the learning epoch, the network has specialized with 290 neurons sensitive to the first correlated pool and the 210 remaining neurons sensitive to the second correlated pool **B,C:** Connectivity matrices for the **B** input and **C** recurrent weights (only 100 of each group are represented for clarity purpose) at the end of the learning epoch, where darker pixels indicate stronger weights. Among recurrent connections, the within-group connections were potentiated while the between-group connections remained weak.(EPS)

Figure S7 **Distribution of the firing rates for the network neurons corresponding to** **Fig. 9** **in the main text.** The same color coding applies: solid curve for log-STDP and dashed for mlt-STDP; red for correlated inputs and purple for uncorrelated inputs.
(EPS)
